# Late Suppers

**Published:** 1876-09

**Authors:** 


### *LATE SUPPERS.*

It is a popular notion that the mission of HALL'S JOURNAL OF HEALTH is to advocate the use of bran bread and other trash containing the smallest amount of nutriment.

We recently read an article in a newspaper in which the *untimely* death of Dr. Hall was attributed to "low diet." Now Dr. Hall was mortal, like other men, but by the exercise of prudence in all things, he attained almost three score and ten years, in spite of a naturally frail physical organization, and during the last half of his life was never ill an hour. He attributed his excellent health to his temperate habits, his care in adapting his dress to the changes of temperature—and, most incredible of all—to a generous indulgence at the table, in the best and the greatest variety of wholesome foods the market afforded.

The immediate cause of his death will never be known, since, from apparently the most perfect health, he was stricken down suddenly, and no autopsy was made ; but it is preposterous to suppose that his death was caused by innutrition, or, to use a plainer word—starvation. He may have said, as the article we refer to charges, "that nothing should be eaten after three o'clock P. M. until the next morning ;" but he evidently meant to say that the "last *hearty* meal of the day should not be taken later than three o'clock." We know, in fact, that this is what he meant, for he always took his own supper as late as six P. M., and nothing after that. He believed it detrimental to health to do so. Although agreeing in most matters relating to health, we differed here.

We contend that it is the abuse of these things that causes the mischief. A glutton is no more a glutton at night than in the morning. It is his excesses on all occasions that destroy his health, and make him repulsive to others. But it is contrary to reason and to the teachings of nature to say that we should not appease hunger regardless of the hour. Food is fuel. If insufficient, there is feeble and unhealthy action, and the machine soon stops.

If we wish to keep the house warm and comfortable, we must replenish the fires before they burn out.

There is amazing ignorance among that peculiar class of people in this country, particularly in large cities, who consider themselves the aristocracy, in regard to the proper care of their children; and, as a consequence, these children are generally inferior mentally and bodily to the children of less pretentious families. Nine years ago a gentleman and his wife, and child of three years, were spending the summer at a country hotel near New York. The parents through mistaken kindness to their child, starved it, until its sufferings excited the sympathies of several of the boarders who had observed its condition, and they endeavored to reason with the parents. This was regarded as an impertinent interference, and the only thing that could be done under the circumstances, was to give the child food whenever there was an opportunity to do so. This plan was soon discovered and frustrated by the parents, who seemed to regard it as a conspiracy against the life of their famishing child. Sensible people predicted that the child would soon become a permanent invalid under such cruel treatment. I met the father a few days since for the first time in years. I inquired after his little daughter. He answered, with a sigh, "She is suffering from a nervous disease, from which I fear she will never recover."

The child's health was destroyed through ignorance, but when it dies it will be called a dispensation of Providence. In our native village there were, perhaps, five families, averaging five children in each family, who attended the same school and were friends and companions. We felt as free in any of these homes as in our own. If we were hungry we knew the way to five well stocked pantries; and if we happened in at meal time, it was not necessary to wait for an invitation to sit down to the table. The invitation was permanent. We never experienced the cravings of hunger for any longer time than was necessary to reach the nearest house, it mattered not what hour of the day or evening. We never heard anything about dyspepsia, or the injurious effects of over eating. Our parents would as soon have thought of going to the pasture fields and warning the horses against eating too much grass, as to have confronted us with any of this modern nonsense. For more than twenty years was this sort of *dissipation* kept up, and we all lived through it. During that time there was not to my recollection a day's serious sickness in the five families, and not a death. There was but one doctor in the village, and he owned a farm, which was about his *only* support. Among those children I believe all but two are living and in good health. The two lived to manhood. Our experience will be found to compare with that of most men who have been raised in "well-to-do" families in country villages. These people possess good, strong common sense, and are hampered by no absurd theories about the kind or quantity of their food and the time to take it. They eat whenever they are hungry, of whatever they relish, and usually stop eating when their hunger is satisfied. The ranks of the best business men in every city are recruited from the country. These recruits make the strongest spokes in the wheels of commerce.

